# Reliability and validity of the Health‐Related Quality of Life Instrument with 8 Items for measuring health‐related quality of life in family caregivers of people with dementia

**DOI:** 10.1002/alz.083961

**Published:** 2025-01-09

**Authors:** Yu Mi Choi, Hyun‐Ju Seo, Seong Min Kim

**Affiliations:** ^1^ Chungnam National University, Daejeon Korea, Republic of (South); ^2^ Chungnam National University, Daejeon, Daejeon Korea, Republic of (South); ^3^ Dowool Health Welfare Center, Namwon Korea, Republic of (South)

## Abstract

**Background:**

Caring for people with dementia can be demanding and challenging, requiring constant support and attention. Family caregivers often experience high‐stress levels, depression, and overall strain. To enhance the quality of life of family caregivers, it is crucial to use accurate measurement tools to identify their needs.

It is unclear whether the Health‐Related Quality of Life Instrument with 8 Items (HINT‐8) is suitable for measuring the HRQoL of Korean family caregivers of people with dementia, as no studies have been conducted to assess its applicability.

This study aims to assess the reliability and validity of the HINT‐8 tool compared with EQ‐5D‐5L and WHOQOL‐BREF in measuring the HRQoL of family caregivers of people with dementia in Korea.

**Method:**

This study is designed as a methodological study, using a cross‐sectional design to verify the reliability and validity of HINT‐8. The Three HRQoL measures ‐ HINT‐8, EuroQoL 5‐Dimension 5‐Level (EQ‐5D‐5L), and WHOQOL‐BREF were provided to 340 family caregivers with people with dementia in dementia relief centers and home‐visiting nursing centers in Korea. The HRQoL scores obtained using the HINT‐8 were evaluated for subgroups with known differences based on demographics and recipient‐related characteristics (known‐group validity). Convergent and divergent validity to assess the Correlation coefficients of the HINT‐8 with other instruments and the intra‐class correlation coefficient (ICC) for test‐retest reliability were evaluated.

**Result:**

The study will calculate descriptive statistics (including median, first quartile, third quartile, minimum, maximum, mean, and standard deviation) for all responses. We will also compare weighted percentages to examine ceiling effects for each HRQoL instrument. We will present the results of the construct validity, test‐retest reliability, and known‐group validity of HINT‐8.

**Conclusion:**

This study will show the validity and reliability of HINT‐8 for measuring health‐related quality of life in family caregivers of people with dementia in a Korean context.

